# Immunomodulatory Protein from *Nectria haematococca* Induces Apoptosis in Lung Cancer Cells via the P53 Pathway

**DOI:** 10.3390/ijms20215348

**Published:** 2019-10-28

**Authors:** Jing-Jing Wang, Yan Wang, Lizhen Hou, Fengjiao Xin, Bei Fan, Cong Lu, Lijing Zhang, Fengzhong Wang, Shuying Li

**Affiliations:** Institute of Food Science and Technology, Chinese Academy of Agricultural Sciences, No. 2 Yuan Ming Yuan West Road, Beijing 100193, China; wangjingjing0536@gmail.com (J.-J.W.); wangyan@caas.cn (Y.W.); houlizhen666@gmail.com (L.H.); xinfengjiao@caas.cn (F.X.); fanbei@caas.cn (B.F.); lucong@caas.cn (C.L.); zhanglijing@caas.cn (L.Z.)

**Keywords:** fungal immunomodulatory protein, *Nectria haematococca*, A549, NMR metabolomics

## Abstract

Our previous research has shown that a fungal immunomodulatory protein from *Nectria haematococca* (FIP-nha) possesses a wide spectrum of anti-tumor activities, and FIP-nha induced A549 apoptosis by negatively regulating the PI3K/Akt signaling pathway based on comparative quantitative proteomics. This study further confirmed that the anti-lung cancer activity of FIP-nha was significantly stronger than that of the reported LZ-8 and FIP-fve. Subsequently, ^1^H NMR-based metabolomics was applied to comprehensively investigate the underlying mechanism, and a clear separation of FIP-nha-treated and untreated groups was achieved using pattern recognition analysis. Four potential pathways associated with the anti-tumor effect of FIP-nha on A549 cells were identified, and these were mainly involved in glycolysis, taurine and hypotaurine metabolism, fructose and mannose metabolism, and glycerolipid metabolism. Metabolic pathway analysis demonstrated that FIP-nha could induce A549 cell apoptosis partly by regulating the p53 inhibition pathway, which then disrupted the Warburg effect, as well as through other metabolic pathways. Using RT-PCR analysis, FIP-nha-induced apoptosis was confirmed to occur through upregulation of p53 expression. This work highlights the possible use of FIP-nha as a therapeutic adjuvant for lung cancer treatment.

## 1. Introduction

Lung cancer is the most common type of cancer and the leading cause of cancer-related death among both men and women. Its incidence accounts for approximately 17.09% of all cancer cases, while the death rate accounts for approximately 21.68% of all cancer-related deaths [1]. Non-small cell lung cancer (NSCLC) accounts for approximately 80% of all lung cancer cases. Only 15% of patients diagnosed with NSCLC survive longer than five years, generally due to late diagnosis and metastasis [2]. Treatment for advanced NSCLC commonly includes chemotherapy, and most patients can easily develop drug resistance [3]. Once metastasis or drug resistance develops, there are few effective treatments available. Therefore, novel drugs and strategies are needed to overcome lung cancer in order to obtain better prognoses.

Fungal immunomodulatory proteins (FIPs) are a protein family first identified in *G. lucidum* in 1989 [4]. In addition to anti-tumor activities, FIPs exhibit direct cytotoxicity to cancer cells by activating the immune system. Moreover, FIPs also show direct cell toxicity against drug-resistant tumor cells [5,6]. FIPs inhibit tumor growth through a variety of effects, such as apoptosis, autophagy, premature senescence, and cell cycle arrest [7,8]. FIPs have been shown to exhibit several inhibitory effects on NSCLC and multidrug-resistant lung cancer cells. However, clear evidence of the mechanisms by which FIPs regulate NSCLC death remains scarce. In previous studies, rLZ-8 and rFIP-fve have been used as positive and negative controls in assessing tumor resistance, respectively. Comparative structural analysis indicated that the difference in the biological activity of these FIPs was due to significant local conformational changes at the two loop regions (Loop DE and Loop FG) of the fibronectin type III (FNIII) domain, which may be a potential active site [9,10].

Metabolic change is a hallmark of cancer that has recently attracted great attention [11]. Metabolomics refers to the study of metabolic processes in biological systems, and primarily aims to identify metabolic biomarkers for diagnostics or pathological identification or to clarify the mechanisms associated with a specific biochemical event [12,13,14]. Nuclear magnetic resonance (NMR), a high-throughput testing technology, is widely used along with multivariate statistics in metabolomic studies. Compared to other analytical techniques, NMR has already proven highly useful for identifying cancer biomarkers and monitoring pharmacodynamic effects [15,16,17,18].

In preliminary studies, we identified a FIP from *N. haematococca*, FIP-nha, that had a strong anti-tumor effect against the human leukemia HL60, gastric cancer MGC823, and liver cancer HepG2 cell lines by activating cell death via apoptosis, and these apoptotic effects were cell-type specific [19]. By conducting comparative quantitative proteomics data analysis, we found that FIP-nha suppressed the growth of human NSCLC A549 cells by negatively regulating the PI3K/Akt signaling pathway [20]. In this study, combined with previous studies, the anti-tumor capacity and effect of rFIP-nha on A549 cells was further investigated. Based on the complexities of the crosstalk among anti-tumor pathways, metabolomics was used to systemically study the metabolic response of A549 cells to rFIP-nha and to determine the potential regulatory mechanism.

## 2. Results

### 2.1. Recombinant FIP-Nha (rFIP-nha) has Stronger Antitumor Capacity Than the Widely Studied FIPs

**Figure 1:**
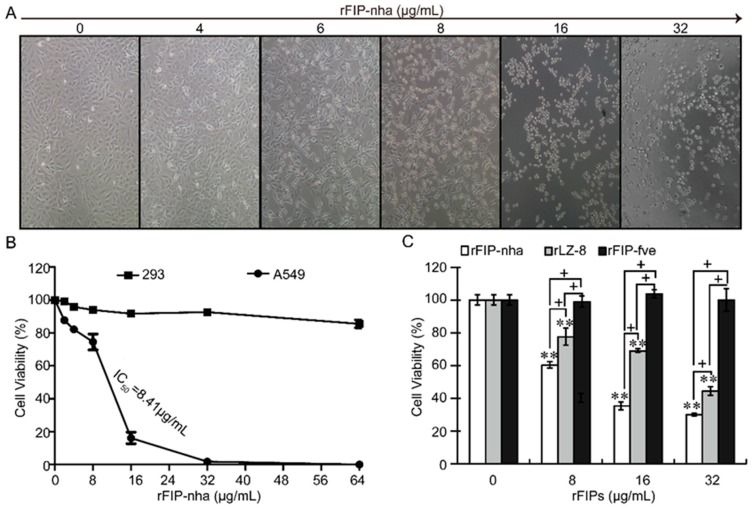
Cytotoxicity assay of recombinant fungal immunomodulatory protein from *Nectria haematococca* (rFIP-nha) in A549 cells. (**A**) Morphological observation of A549 cells after rFIP-nha treatment. A549 cells were treated with 0, 4, 6, 8, 16, or 32 μg/mL rFIP-nha for 24 h, and micrographs were collected on a Zeiss microscope at 200 × magnification. (**B**) Cell viability assay. A549 cells and normal HEK293 cells were treated with varying concentrations (0, 2, 4, 8, 16, 32, or 64 μg/mL) of rFIP-nha for 24 h, followed by CCK assays. (**C**) Comparison of cytotoxicity between rFIPs. A549 cells were treated with varying concentrations (0, 8, 16, or 32 μg/mL) of rFIP-nha, rLZ-8, or rFIP-fve for 24 h, followed by CCK assays to estimate cell viability. Each bar represents mean ± SD (*n* = 3). **: *p* < 0.01 compared to control (0 μg/mL). +: *p* < 0.05 between different FIPs at the same concentration.

LZ-8 (FIP-glu) from *G. lucidum* and FIP-fve from *Flammulina velutipes* are two of the earliest FIPs identified and the most widely studied; LZ-8 has significant direct anti-tumor activity, whereas FIP-fve has extremely weak direct anti-tumor activity [21,22]. In evaluating tumor resistance capacity, rLZ-8 and rFIP-fve have been used as the positive and negative controls for investigating the proliferation inhibitory effect of rFIP-nha on A549 cells (Figure 1C). Compared to untreated cells, the cytotoxicity of rFIP-nha and rLZ-8 was clear (*p* < 0.01); however, the viability of A549 cells in the presence of rFIP-fve was almost unchanged. A comparative study of rFIP-nha and rLZ-8 in A549 cells indicated that the cytotoxicity of rFIP-nha was much higher than that of rLZ-8 (*p* < 0.05). These results confirmed that rFIP-nha is a potent tumor inhibitor in NSCLC cell lines, with great potential in the clinic.

When compared with untreated cells, cell viability decreased sharply with increased concentrations of rFIP-nha (*p* < 0.01), and the calculated IC_50_ value was approximately 8.41 μg/mL, but rFIP-nha treatment had milder effects on normal human embryonic kidney 293 (HEK293) cells (Figure 1B). Treatment with rFIP-nha for 24 h caused obvious morphological changes in A549 cells in a concentration-dependent manner. A549 cells gradually shrank and became rounded as the concentration (0, 4, 6, 8, 16, and 32 μg/mL) of rFIP-nha increased. When the concentration of rFIP-nha was higher than 8 μg/mL, the A549 cells were completely shrunken and rounded (Figure 1A).

### 2.2. Metabolite Profile Analysis by ^1^H NMR Metabolomics

**Figure 2:**
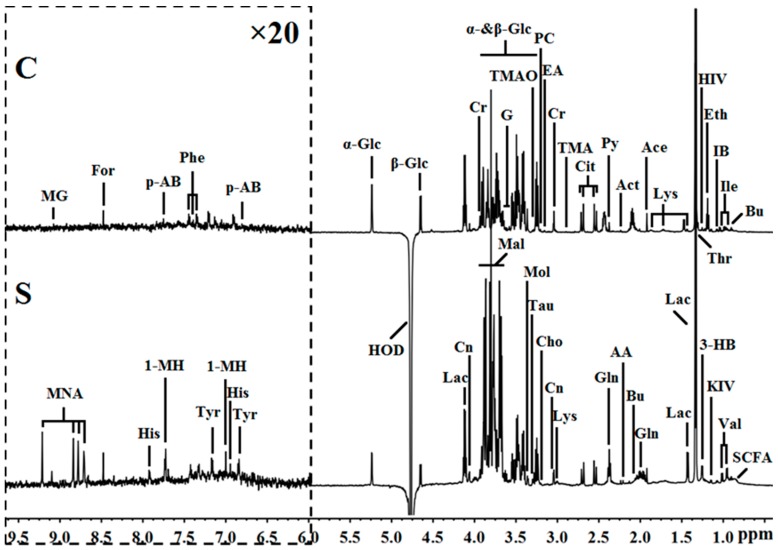
Typical ^1^H NMR spectra (δ0.5-9.0) of A549 cell extracts obtained from groups treated with (**S**) and without (**C**) rFIP-nha. The region of δ6.0–9.5 (in the dashed box) was magnified 20 times compared with the corresponding region of δ0.5–6.0 for clarity. Keys: 1-MH, 1-methylhistideine; 3-HB, 3-hydroxybutyrate; AA, acetoacetate; Ace, acetate; Act, acetone; Ala, alanine; Bu, butyrate; Cho, choline; Cit, citrate; Cn, creatinine; Cr, creatine; EA, ethanolamine; Eth, ethanol; For, formate; G, glycerol; Glc, glucose; Gln, glutamine; His, histidine; HIV, 3-hydroxyisovalerate; HOD, the residual water signals; IB, isobutyrate; Ile, isoleucine; KIV, 2-ketoisovalerate; Lac, lactate; Lys, lysine; Mal, mannitol; MG, 7-methylguanosine; MNA, 1-methylnicotinamide; Mol, methanol; p-AB, p-aminobenzoate; PC, phosphocholine: Phe, phenylalanine; Py, pyruvate; SCFA, short-chain fatty acid; Tau, Taurine; Thr, threonine; TMA, trimethylamine; TMAO, trimethylamine N-oxide; Tyr, tyrosine; Val, valine.

The above results confirmed that rFIP-nha had stronger cytotoxicity towards A549 cells than those of the reported rLZ-8 and rFIP-fve. Morphologically, after A549 cells (2 × 10^6^) were treated with 16 μg/mL of rFIP-nha, cells completely shrank and became rounded; therefore, this concentration was chosen for deep mechanistic exploration. ^1^H NMR metabolomics was used to analyze the metabolite profile changes because of its simple and fast sample preparation, short analysis times, and low cost of analysis [23]. Typical ^1^H NMR spectra of A549 cells treated with rFIP-nha (S) and untreated cells (C) are shown in Figure 2. Primary resonances in the ^1^H NMR spectra were assigned to individual metabolites according to date from previous study [18] and were confirmed by the Human Metabolome Database version 3.6 (http://www.hmdb.ca/). Then, multivariate data analysis was employed to identify the differences.

**Figure 3:**
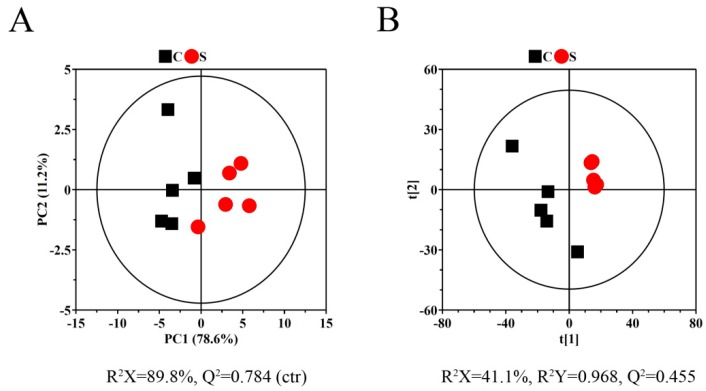
(**A**) 2D principal component analysis (PCA) scores plots based on ^1^H NMR spectra of A549 cells obtained from groups C and S. (**B**) Partial least squares-discriminant analysis (PLS-DA) scores plots based on ^1^H NMR spectra of A549 cells obtained from groups C and S. The experimental group of A549 cells (group S) were treated with 16 μg/mL FIP-nha for 24 h, and the control group (group C) was treated with same volume of PBS for 24 h.

Unsupervised principal component analysis (PCA) and supervised partial least squares-discriminant analysis (PLS-DA) were used as multivariate analysis methods. PCA and PLS-DA score plots constructed with ^1^H NMR spectral data from A549 cell samples were used to depict the general variation between treated and untreated groups with rFIP-nha (Figure 3). The rFIP-nha treatment group was completely distinct from the control group. These results suggested that rFIP-nha could inhibit the pathological process of A549 cells and effectively normalize their metabolic perturbations.

**Figure 4:**
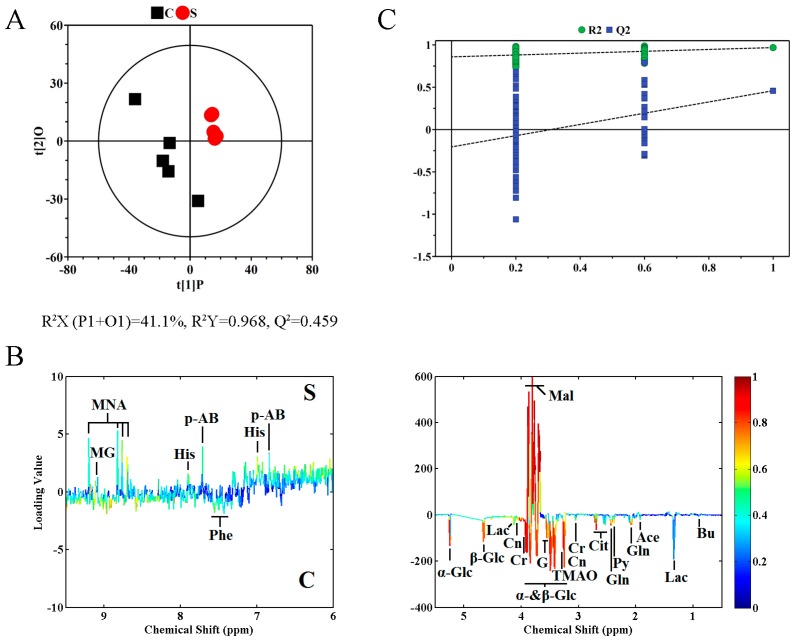
Orthogonal projection to latent structure with discriminant analysis (OPLS-DA) scores plots (**A**) derived from ^1^H NMR spectra of A549 cells. Corresponding coefficient loading plots (**B**) obtained from groups C and S and cross validation (**C**) by permutation test (*n* = 200). The significance of metabolites variations between the two classes is shown in a color map. Peaks in the negative direction demonstrate metabolites of group C are more abundant. In the group S, if metabolites are more abundant, the peaks will in the positive direction. Figure 2 shows the keys of the assignment.

To identify differential metabolites, we performed the orthogonal projection to latent structure with discriminant analysis (OPLS-DA) on corresponding NMR data. In Figure 4, the OPLS-DA score and the corresponding loading plots are shown. Table 1 shown the biochemical alterations in A459 cell treated with rFIP-nha, including differential metabolites with significant correlation coefficients between groups. A permutation test (permutation number = 200) was conducted in order to assess the validity of these models; meanwhile, five-fold cross-validation parameters, Q2 and R2, were calculated based on the corresponding PLS-DA model in Figure 4.

Nineteen significantly altered metabolites were selected based on the OPLS-DA coefficients (|FC| > 0.5) and VIP values (VIP > 1) (Table 1). Compared with the untreated cells, A549 cells treated with rFIP-nha showed a significant increase in 1-methylnicotinamide, 7-methylguanosine, histidine, mannitol, p-aminobenzoate, and significant decreases in acetate, butyrate, citrate, creatine, creatinine, glutamine, glycerol, lactate, phenylalanine, pyruvate, taurine, trimethylamine N-oxide, α-glucose, and β-glucose.

### 2.3. Metabolic Pathway Analysis

**Figure 5:**
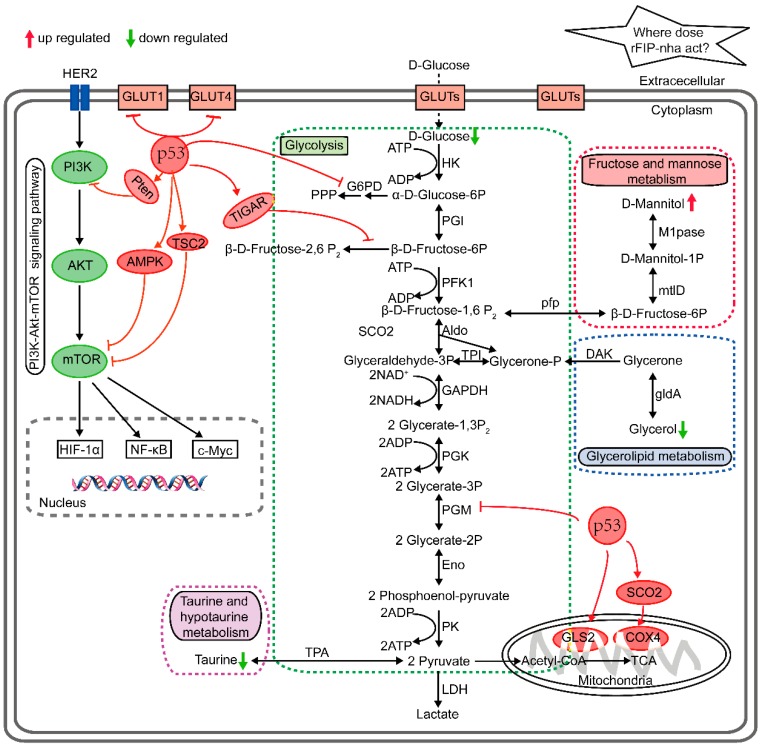
Summary of altered metabolic pathways and regulatory mechanism of rFIP-nha against A549 cells. HK, hexokinase; PGI, phosphoglucose isomerase; PFK1, phosphofructokinase 1; Aldo, aldolase; TPI, triose phosphate isomerase; GAPDH, glyceraldehyde 3-phosphate dehydrogenase; PGK, phosphoglycerate kinase; PGM, phosphoglycerate mutase; Eno, enolase; PK, pyruvate kinase; LDH, lactate dehydrogenase; TCA, tricarboxylic acid cycle; G6PD, glucose-6-phosphate dehydrogenase; TPA, taurine-pyruvate aminotransferase; pfp, pyrophosphate-fructose-6-phosphate 1-phosphotransferase; mtlD, mannitol-1-phosphate 5-dehydrogenase; M1Pase, mannitol-1-phosphatase; gldA, glycerol dehydrogenase; DAK, dihydroxyacetone kinase; GLUTs, glucose transporters; HER2, receptor tyrosine-protein kinase erbB-2; PI3K, phosphatidylinositol 3-kinase; Akt, serine-threonine kinase; mTOR, mammalian target of rapamycin; HIF-1α, hypoxia-inducible factor 1α; NF-κB, nuclear factor kappa-light-chain-enhancer of activated B cells; c-Myc, a transcription factor; p53, a tumor suppressor; Pten, a human tumor suppressor gene on chromosome 10; AMPK, AMP-activated protein kinase; TSC2, tuberous sclerosis complex 2; TIGAR, TP53-induced glycolysis and apoptosis regulator; GLS2, glutaminase 2; SCO2, cytochrome c oxidase 2; COX4, cytochrome c oxidase subunit 4. HER2-mediated PI3K-Akt-mTOR signaling plays a pivotal role in promoting glycolysis in tumor cells via the activation of HIF-1α, NF-κB, and c-Myc. p53 plays a key role in the process of suppressing glycolysis and promoting oxidative phosphorylation (OXPHOS) by interacting with various enzymes and other molecules, including SCO2, TIGAR, GLUT1, GLUT4, GLS2, and PGM. Different metabolic pathways represented within the dashed box. The arrow represents stimulatory modification, the T-shaped arrow represents inhibitory modification.

The most relevant pathways in A549 cells treated with rFIP-nha were explored using metabolic pathway analysis (MetPA) with MetaboAnalyst 3.0 (http://www.metaboanalyst.ca/) and the published literature [24,25]. The disturbed metabolic pathways and the differential metabolites are shown in Figure 5. These metabolites were involved in four metabolic pathways: Glycolysis (glucose), taurine and hypotaurine metabolism (taurine), fructose and mannose metabolism (mannitol), and glycerolipid metabolism (glycerol). These might be the target pathways by which rFIP-nha acts on A549 cells.

### 2.4. Validation of P53 Expression

**Figure 6:**
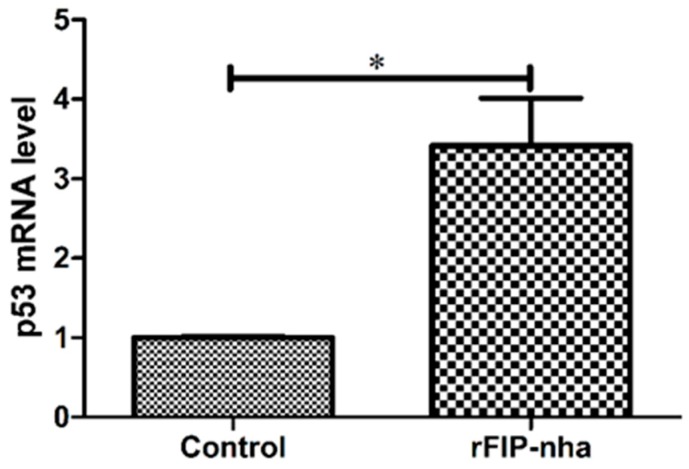
Analysis of differential p53 expression in A549 cells after rFIP-nha treatment. A549 cells were treated with 16 μg/mL of rFIP-nha for 24 h, followed by RT-PCR assays to estimate p53 expression changes. Each bar represents mean ± SD (*n* = 3); *: *p* < 0.05 compared to control (0 μg/mL).

The multifaceted regulator p53 plays a pivotal role in the regulation of cancer metabolism by activating metabolism-related enzymes, regulating the expression of specific genes, and engaging in crosstalk with other key factors to inhibit multiple oncogenic processes [24,25]. Therefore, we further characterized apoptosis in A549 cells by detecting p53 expression with and without rFIP-nha treatment (Figure 6). The test results showed that after treatment with 16 μg/mL rFIP-nha, p53 mRNA expression levels in A549 cells were more than doubled.

## 3. Discussion

A dozen FIPs have now been identified, but only a few of them have been extensively studied with respect to their anti-tumor activities [8]. These FIPs can be simply divided into two groups: One group comprises the FIPs from *Ganoderma* spp., and the other includes FIP-fve from *F. velutipes*. Investigation of their anti-tumor activities indicated that the FIPs from *Ganoderma* spp. have strong, direct anti-tumor activities; whereas the direct anti-tumor effects of FIP-fve are weak. Normally, FIPs from *Ganoderma* spp. at 8 μg/mL show obvious anti-tumor activity towards human tumor cells [21,26], but FIP-fve did not show anti-tumor activity until the concentration reached 195 μg/mL [22]. In addition to the above FIPs, five other FIPs with excellent direct anti-tumor effects have been identified by our laboratory using genetic engineering methods [20,27,28,29]. Among them, FIP-nha showed the strongest and broad-spectrum anti-tumor activities, and could induce A549 cell apoptosis by G1/S arrest [20]. In this study, we further confirmed that rFIP-nha was significantly toxic to A549 cells but had no effects on normal HEK293 cells. Moreover, the cytotoxicity of rFIP-nha towards A549 cells was obviously higher than that of rLZ-8 or rFIP-fve.

The anti-tumor effects of FIPs from different sources vary greatly. FIPs from *Ganoderma* spp. shared considerably higher sequence, structural and activity similarity, but their anti-tumor effects are variable. Thus far, four FIPs from *Ganoderma spp.*, LZ-8 (*G. lucidum*), FIP-gts (*G. tsugae*), FIP-gmi (*G. microsporum*). and FIP-gsi (*G. sinensis*) have exhibited strong direct anti-tumor activity. rLZ-8 has been shown to inhibit the proliferation of human glioblastoma and chronic myeloid granulocyte leukemia cells by inducing apoptosis [30]. FIP-gts inhibits A549 cell growth, leading to G1 arrest, consequently inducing premature senescence [31]. Additionally, FIP-gts induces autophagic cell death against drug-resistant urothelial cancer cells [5]. FIP-gmi inhibits tumor growth and significantly induces autophagy, but not apoptosis, in NSCLC and multidrug-resistant lung cancer cells [6,26]. FIP-fve suppresses cell proliferation by inducing cell cycle arrest but does not induce apoptosis [22].

Based on the consistent knowledge of anti-tumor effects (apoptosis, autophagy, premature senescence), A549 was selected as the cell model, and several key genes were selected to explore the anti-tumor mechanisms of FIPs through RT-PCR and Western blot assays. FIP-gts exhibits anti-tumor activity by regulating telomerase expression [32] and inhibits telomerase activity in lung cancer cells through nuclear export mechanisms and ER stress-induced intracellular calcium levels [33]. FIP-gmi induces autophagy through the Akt-mTOR-p70S6K pathway, thereby inhibiting multidrug-resistant lung cancer cells [6]. FIP-fve suppresses lung cancer cell proliferation via p53 activation [22].

In early studies, we confirmed that FIP-nha induced A549 cell apoptosis by negatively regulating PI3K/Akt signaling using comparative quantitative proteomics [20]. Because of the complexities of antitumor effects and crosstalk among anti-tumor pathways, ^1^H NMR-based metabolomics was applied to investigate A549 cell metabolic signatures after rFIP-nha treatment with the aim of comprehensively understanding the underlying mechanisms and assessing the diagnostic potential of this protein. Our researched confirmed that 19 metabolites were significantly altered. On analyzing the metabolic pathways, glycolysis (glucose), taurine and hypotaurine metabolism (taurine), fructose and mannose metabolism (mannitol), and glycerolipid metabolism (glycerol) were found to be related to the process.

Aberrant glucose metabolism, ”aerobic glycolysis” (the Warburg effect), is a hallmark of human cancers, and it increases the synthesis of macromolecules and intermediates to maintain tumor growth and proliferation [25]. A cluster of ”multifaceted regulators” are involved in this process. These multifaceted regulators modulate the crucial transcription factors or metabolic enzymes involved in glycolysis and oxidative phosphorylation (OXPHOS) and can efficiently modulate glucose metabolism and enhance tumor cell survival. Additionally, some metabolism-related pathways are also crucial for cancer glucose metabolism, especially the PI3K-AKT-mTOR and AMPK pathways [24].

The multifaceted regulator p53, a tumor suppressor, plays a central role in cancer glucose metabolism [34,35,36,37]. First, p53 represses aerobic glycolysis by regulating glucose transporters. p53 directly suppresses the expression and translocation of glucose transporter 1 (GLUT1) and GLUT4 [38] and indirectly downregulates GLUT3 [39] to reduce glucose uptake from the tumor microenvironment. Second, p53 regulates glucose metabolism through the regulation of metabolic enzymes. p53 induces the transcription of TIGAR (TP53-induced glycolysis and apoptosis regulator), which dephosphorylates fructose-2,6-bisphosphate to fructose-6-phosphate and diverts glucose catabolism to the pentose phosphate pathway (PPP) [40,41]. Further, p53 also inhibits glucose-6-phosphate dehydrogenase (G6PD), the rate-limiting enzyme in the PPP [21]. Moreover, p53 promotes the ubiquitination-mediated degradation of phosphoglycerate mutase (PGM) and prevents the conversion of fructose-1,6-bisphosphate to pyruvate [42]. In OXPHOS, p53 increases the transcription of cytochrome c oxidase 2 (SCO2) and glutaminase 2 (GLS2), which increases the activity of the TCA cycle and the rate of OXPHOS [43,44]. p53 also inhibits glycolysis by inducing a group of target genes to negatively regulate the PI3K-AKT-mTOR pathway. For example, p53 induces Pten to inhibit PI3K-AKT signaling [45] and activates AMP-activated protein kinase (AMPK) and tuberous sclerosis complex 2 (TSC2) to negatively regulate mTOR activity [45,46].

Based on the data from this study and the above literature related to the mechanisms of the anti-tumor and cancer glucose metabolism effects, we propose that rFIP-nha induces A549 cell apoptosis at least partially by modulating the multifaceted roles of p53, which then inhibits glycolysis and facilitates the TCA cycle, OXPHOS, and other metabolic pathways (Figure 5). Analysis of p53 expression confirmed the reliability of our hypothesis and implied that the potential targets of rFIP-nha are located proximal to p53 (Figure 6).

These conclusions are currently speculative, and determination of the actual mechanisms requires further validation. In the future, transcriptomics should be further explored to determine the key genes of FIP-nha-induced A594 apoptosis, and multi-omics combined analysis should be performed to find the target pathways. Furthermore, the potential targets of rFIP-nha remain unknown. Determining the interaction partners of rFIP-nha and in-depth elucidation of the role of ”multifaceted regulators” in inhibiting cancer glucose metabolism will provide novel insights for anticancer research and offer promising therapeutic targets for FIP-nha in anti-cancer therapies.

In conclusion, the data from this study indicated that rFIP-nha exhibit a stronger antitumor capacity than the previously reported FIPs, rLZ-8 and rFIP-fve. The metabolic profile changes in A549 cells after rFIP-nha treatment demonstrated that the apoptosis-associated cell death resulted from rFIP-nha-induced p53 activation, which then resulted in inhibition of the PI3K-Akt-mTOR and/or AMPK pathway and subsequent metabolic disturbance of A549 cells. The effects of rFIP-nha on A549 cell apoptosis provide a better understanding regarding the roles of rFIP-nha in lung cancer therapy and indicate its potential as a candidate for treating NSCLC.

## 4. Materials and Methods

### 4.1. Expression and Purification of FIPs

Recombinant FIPs (rFIP-nha, rLZ-8, rFIP-fve) were expressed and purified as previously described [5,19].

### 4.2. Cell Culture

Human lung adenocarcinoma A549 cells and embryonic kidneyHEK293 cells were obtained from the cell resource center of Peking union medical college hospital (Beijing, China). Cells were maintained at 37 °C in a 5% CO_2_ humidified atmosphere in Dulbecco’s Modified Eagle’s Medium (DMEM) (Gibco, Rockville, MD, USA) containing 10% fetal bovine serum (FBS; Life Technologies, Inc., Rockville, MD, USA), 100 units/mL penicillin, and 100 μg/mL streptomycin (Life Technologies, Inc., Rockville, MD, USA).

### 4.3. Tumor Cell Proliferative Inhibitory Assay

Aliquots of 100 µL A549 cell suspension (2.5 × 10^5^ cells/mL) were seeded into a 96-well microplate and cultured for 24 h. Then, 100 µL serially diluted rFIP (rFIP-nha, rLZ-8, or rFIP-fve) was added into the wells of the 96-well microplate and continually cultured for another 24 h. Morphological changes were observed using an inverted microscope (Axiovert 40 CFL, Zeiss, Germany) equipped with a digital camera (AxioCam MRm, Zeiss, Germany). A549 cell viability was analyzed by CCK assays using a TransDetect^TM^ Cell Counting Kit (TransGen Biotech, Beijing, China).

### 4.4. Sample Preparation

A549 cells (2 × 10^6^) were cultured in 60 mm dishes for 24 h and treated with 0 or 16 μg/mL FIP-nha for 24 h. Cells were harvested by centrifugation and washed three times with cold PBS (pH 7.4, 10 mM). The cell pellets were dissolved in 2 mL acetonitrile: Water (1:1), lysed by using an ultrasonic wave for 2 s, and then frozen and thawed twice in liquid nitrogen. The upper phase (aqueous phase) of each sample was collected by centrifuging at 15,000 × g for 10 min at 4 °C and evaporating to dryness under a nitrogen gas stream. Before NMR analysis, the residue was dissolved with 500 μL D_2_O and 50 μL Na salt of 3-trimethylsilyl-2, 2, 3, 3-tetradeutero propionic acid (TSP 1 mg/mL in D_2_O) for a field frequency lock. After centrifugation at 12,000 × g for 10 min, the supernatant was transferred into a 5 mm NMR tube for NMR analysis.

### 4.5. ^1^H NMR Measurements and Spectral Data Processing

All samples were detected by ^1^H NMR spectroscopy at 600.13 MHz using a Bruker AVANCE III 600 spectrometer (Varian Inc., Palo Alto, CA, USA) operating at 298 K. A one-dimensional spectrum was acquired by using a standard PRESAT pulse sequence to suppress the water signal with a relaxation delay of 5 s. Sixty-four free induction decays (FIDs) were collected into 32 K data points with a spectral width of 8000 Hz, an acquisition time of 2.7 s, and a total pulse recycle delay of 2.0 s. Spectra were manually phased and baseline adjusted, and chemical shifts were referenced to the TSP resonance at δ 0.00. The FIDs were multiplied by an exponential weighting function equivalent to a line broadening of 0.5 Hz prior to Fourier transformation and zero-filled by a factor of 2. All the ^1^H NMR spectra had been manually Fourier transformed in MestReNova software (V9.0.1; Mestrelab Research, San Diego, CA, USA), to reduce the complexity of the NMR data. After phase adjustment and baseline correction, the spectrum was divided into 77 segments ranging from 9.5 to 0.5 ppm. The region 5.22–4.67 ppm was removed to eliminate baseline effects of imperfect water saturation and the broad resonance from cells, both of which represent highly variable regions in the spectra.

### 4.6. NMR Metabolomics Data Analysis with MetaboAnalyst

NMR data were analyzed using MetaboAnalyst 3.0 (http://www.metaboanalyst.ca/). The NMR data contain a data matrix of 10 samples (5 controls and 5 samples treated with rFIP-nha) by 77 spectral bins. Eight variables were removed for a threshold of 50%. Variables with missing values were replaced with a small value (half of the minimum positive value in the original data). Five percentage features were reduced based on interquartile range after data filtering. The normalization consisted of the following operations: Row-wise normalization; normalization to constant sum; data transformation, N/A; and data scaling, auto scaling. Unpaired fold change (FC) analysis and *t*-tests were used as univariate analysis methods. Principal component analysis (PCA) and partial least squares-discriminant analysis (PLS-DA) were chosen as the multivariate analysis methods. Important features were selected by fold-change analysis with a threshold of 1.5, *t*-tests with a threshold of 0.05, and a VIP score of component 1 with a threshold of 1 in PLS-DA. Pathway analysis was also conducted using MetaboAnalyst 3.0. The selected pathway library is *Homo sapiens* (human). The selected over-representation analysis method was the hypergeometric test, and the selected node importance measure for topological analysis was relative betweenness centrality.

### 4.7. Real-Time PCR Assay for P53 Expression

p53 expression in A549 cells after treatment with 16 μg/mL rFIP-nha was evaluated by quantitative real-time PCR (qPCR), and untreated A549 cells were used as a control. Total RNA was isolated from approximately 2 × 10^5^ cells from each sample and reverse transcribed using the SuperScript^®^ III Reverse Transcriptase kit (Life Technologies, AB & Invitrogen, Carlsbad, CA, USA). The designed primer sequences were as follows: p53 forward primer, 5′-GAA GAG ATG GGG GAG GGA GGC TGT CA-3′/p53 reverse primer 5′-GCT CCG GGG ACA CTT TGC GTT CG-3′; actin forward primer, 5′-ATG GGT CAG AAG GAT TCC TAT GT-3′/actin reverse primer 5′-AAG GTC TCA AAC ATG ATC TGG G-3′. SYBR Green I was used as a fluorescent dye to detect the PCR products. The reactions were monitored using an RT-PCR instrument (ABI 7500, Applied Biosystem, Foster City, CA, USA). The mRNA levels were quantified relative to endogenous actin as a control. The 2^−^^ΔΔCt^ method was used for the relative quantification of gene expression.

### 4.8. Statistical Analysis

The experiments were performed at least three times. The data are expressed as the mean values of three replicates and the standard deviation (mean ± SD). The results are presented as a percentage of the negative control (cells without treatment). One-tailed unpaired Student’s *t*-tests were used to compare differences between samples and controls. The differences were considered significant when *p* < 0.05 and extremely significant when *p* < 0.01.

## Figures and Tables

**Table 1:** OPLS-DA coefficients and VIP values derived from the NMR data of A549 cells obtained from groups C and S.

Metabolite	r ^1^	VIP ^2^
1-Methylnicotinamide: 8.69(m), 8.76(m), 8.82(d), 9.20(s)	0.609	1.294
7-Methylguanosine: 9.08(s)	0.501	1.080
Acetate: 1.92(s)	−0.572	1.330
Butyrate: 0.90(t), 2.14(t)	−0.633	1.411
Citrate: 2.54(d), 2.70(d)	−0.856	1.802
Creatine: 3.04(s), 3.93(s)	−0.885	1.856
Creatinine: 3.05(s), 4.06(s)	−0.682	1.496
Glutamine: 2.09(m), 2.43(m), 3.78(t)	−0.833	1.732
Glycerol: 3.58(m), 3.64(m), 3.81(m)	−0.682	1.438
Histidine: 6.98(s), 7.91(s)	0.675	1.406
Lactate: 1.33(d), 4.12(q)	−0.672	1.399
Mannitol: 3.68(dd), 3.76(m), 3.81(d), 3.88(dd)	0.913	1.899
p-Aminobenzoate: 6.83(d), 7.71(d)	0.676	1.493
Phenylalanine: 7.33(d), 7.37(t), 7.43(m)	−0.632	1.372
Pyruvate: 2.38(s)	−0.658	1.440
Taurine: 3.27(t), 3.43(t)	−0.516	1.224
Trimethylamine N-oxide: 3.27(s)	−0.867	1.804
α-Glucose: 3.42(t), 3.54(dd), 3.71(t), 3.84(m), 5.24(d)	−0.875	1.825
β-Glucose: 3.25(dd), 3.41(t), 3.46(m), 3.49(t), 3.90(dd), 4.65(d)	−0.917	1.907

^1^ Correlation coefficients, positive, and negative signs indicate positive and negative correlation in the concentrations, respectively. The correlation coefficient of│r│ > 0.500 was used as the cutoff value for the statistical significance based on the discrimination significance at the level of *p* = 0.05. ^2^ Variable importance in projection. The VIP values more than 1 were used as the cutoff values for the statistical significance.
